# Labor-force participation and working patterns among women and men who have survived cancer: A descriptive 9-year longitudinal cohort study

**DOI:** 10.1177/1403494820953330

**Published:** 2020-09-04

**Authors:** Birgit Brusletto, Roy A. Nielsen, Harald Engan, Line Oldervoll, Camilla M. Ihlebæk, Nina Helen Mjøsund, Steffen Torp

**Affiliations:** 1 Department of Health, Social and Welfare Studies, Faculty of Health and Social Sciences, University of South-Eastern Norway, Norway; 2 Fafo Institute for Labour and Social Research, Norway; 3 Unicare Rehabilitation, Norway; 4 Department of Public Health and Nursing, Faculty of Medicine and Health Sciences, Norwegian University of Technology and Science, Norway; 5 Department of Public Health Science, Faculty of Landscape and Society, Norwegian University of Life Sciences, Norway; 6 Faculty of Health and Social Work Studies, Østfold University College, Norway; 7 Department of Mental Health Research and Development, Vestre Viken Hospital Trust, Norway

**Keywords:** Cancer survivor, return to work, vocational rehabilitation, registries

## Abstract

**Aims:**

Our aim was to investigate labor-force participation, working hours, job changes, and education over 9 years among persons who have survived more than 10 years after cancer, and compare it to controls.

**Methods:**

Register data on 2629 persons who survived cancer were stratified by gender and compared to data on 5258 matched controls. Persons who survived cancer were aged 30–50 when diagnosed with cancer and had a work contract prior to diagnosis. Descriptive analysis and *t*-tests were performed.

**Results:**

The proportion of female persons who survived cancer in the labor force was reduced from 100% to 83.9% during follow-up, demonstrating a significant difference compared to controls for each year measured. The proportion of male persons who survived cancer dropped from 100% to 84.8%, but was only significantly different compared to controls in 2 years. The proportion of female persons who had survived cancer who worked full-time was lower in all years compared to both controls and male persons who survived cancer; in turn, male persons who had survived cancer worked full-time less than male controls. The proportion of female persons who had survived cancer who worked less than 20 hours per week increased compared to controls. The frequency of change of employer was higher among female persons who survived cancer compared to controls for some years, but no significant differences between male persons who survived cancer and controls were found. Female persons who survived cancer were in education more often than male persons who survived cancer.

**Conclusions:**

**Persons who survived cancer experienced reduced labor-force participation and working hours 9 years after diagnosis, and the reduction was more pronounced for women than for men. Working patterns were also different between genders and between persons who survived cancer and controls.**

## Introduction

In Norway, about 40% of cancer patients are working age (20–59 years), and 71% survive more than 5 years after treatment [1]. Therefore, many cancer survivors have to partly or fully maintain their working roles during cancer treatment and/or return to work (RTW) after treatment.

Several studies report a decrease in work continuance and higher unemployment after cancer [2–4]. Approximately six out of 10 persons who survive cancer (PSC) in Europe and were employed before cancer diagnosis RTW during the first year after diagnosis [5]. However, late effects from the disease and treatment, such as fatigue, cognitive dysfunction, or lymphedema, are frequent, and may make working difficult for many years after treatment due to reduced physical and/or mental functioning, affecting RTW and reducing labor-force participation [6, 7]. Long-term work ability may be threatened [6, 8,9].

Modifications made at work after cancer range from no or a small reduction in working hours to change of duties, employer or occupation [9–11]. Employer support and reintegration into social relationships at work strongly promotes successful RTW after cancer treatment [12–15].

Demographic factors such as age, gender, and education level influence the ability to continue work after cancer [16–19]. In Norway, fewer women than men are employed 5 years after cancer [20] and reducing working hours after cancer are more common among women than men [14].

Adapting to late effects and managing changes by reducing working hours or finding a new job during the RTW process seems to take more effort and time than expected, up to several years [2, 21, 22]. However, most research on labor-force participation after cancer has had only short follow-up after diagnosis [9, 20]. To our knowledge, no Scandinavian controlled RTW studies have been carried out that comprise all cancer types for more than 5 years after cancer.

## Aims

This Norwegian registry study describes the patterns of labor-force participation, working hours, job changes, and education for 9 consecutive years among women and men who have survived cancer and compares those patterns with matched controls.

## Methods

We investigated changes in register data variables for female and male PSC alive 10 years after the cancer diagnosis, for 9 consecutive years (as not all work-related variables in the database were complete for the 10^th^ year).

### Registries

PSC were identified using the Cancer Registry of Norway. Work-related data were from Statistics Norway’s events database, FD-Trygd [20], which collects information from several Norwegian registries [20]: age, gender, work contract, working hours, and employer. Data from these three registries were linked through a personal identification number.

### Material

In the Cancer Registry of Norway, we identified 21,819 new cancer cases diagnosed in 2004 and 2005 for all ages; 12,548 survived for 10 years or more, and 3579 of those were 30–50 years old when diagnosed.

The range 30–50 was chosen based on labor-force participation in general and that cancer is most often diagnosed among people aged 40 and above. Among younger people, many may still be in education in their 20s. Also, younger adults may not have experienced being an employee over a long period of time, which can imply other challenges compared to middle-aged people with longer working experiences. Early retirement increases from about 60 years of age [23, 24] and participants close to this age at the end of follow-up would more likely choose to retire instead of making efforts to RTW.

We excluded those with more than one cancer diagnosis (*n* = 428) because a new cancer diagnosis during a 9-year follow-up might lead to a higher likelihood of quitting work. We also excluded patients without employment within 90 days prior to diagnosis (*n* = 891). Finally, 15 patients were excluded due to missing valid educational codes and 44 persons because they had emigrated. Thus, 2629 PSC were extracted from the Cancer Registry of Norway.

Each PSC was matched with two unique controls randomly extracted from FD-Trygd in 2004/2005, who were alive with no cancer diagnosis between 2004/2005 and 2015. Matching was based on age, gender, education, and being employed, that is, having a work contract. Controls were given a pseudo-diagnosis date according to their corresponding PSC’s time of diagnosis. When controls were matched to a cancer case, they were blocked from being selected again.

The dataset ultimately included 2629 PSC and 5258 controls, who were followed over 2004/2005–2013/2014; all variables were measured every 12^th^ month from date of diagnosis, for both PSC and controls. The year of diagnosis (2004/2005) is labeled T_0_, and so on: T_1_ (2005/2006)–T_9_ (2013/2014).

### Variables

PSCs’ cancer diagnoses were classified using the second topographic level of categorization according to the International Classification of Diseases for Oncology, third edition [25].

*Labor-force participation* was defined as being employed during the 90 days prior to cancer diagnosis and at measurement in a given year thereafter. PSC not employed in a given year were regarded as out of work that year, but were later re-included as employed if a new work contract was registered subsequently.

*Working hours* per week were monitored among the employed each year, divided into three categories: (a) 30 hours or more (full-time), (b) 20–29.9 hours (long part-time), (c) less than 20 hours (short part-time). A normal working week in Norway is 37.5 hours per week.

*Job changes*, conceptually speaking, may include change of duties within the same company as well as change of employer; however, the available dataset provided data only regarding change of employer. Therefore, the variable measured change of employer from 1 year to the next. The year before diagnosis (T_-1_) was not available, and the first year of monitored changes was therefore changed from T_0_ to T_1_.

*Education* was measured by being enrolled as a student at any educational level per October 1 of each year. Because one can take an education while being employed, the total in education was included each year independent of labor-force participation.

### Statistics

Descriptive statistics were performed using frequencies and percentages. Two-sample and paired *t*-tests were used for comparisons between the groups of PSC and controls. All variables were stratified by gender. Stata/SE 14.2 for Windows was used for the analyses. Tables and results from the statistical analyses, confidence intervals and statistical significance, are presented in Supplemental Tables II–IV.

We calculated the proportions of the total sample at the same time each year. All data for variables *labor-force participation, working hours*, and *job changes* were monitored every 12^th^ month; *working hours* and *job changes* were dependent on *labor-force participation*. Significance level was 5%.

### Ethics

The Regional Committees for Medical and Health Research Ethics of Mid-Norway (2016/830) and the Norwegian Data Protection Authority (16/00235) approved the study.

## Results

### Sample characteristics

The population of 2629 PSC consisted of more women (64%) than men; the mean age was 42 years among women and 41 years among men (Table I). The most common diagnosis among women was breast cancer (39%). Among men, the most common diagnosis was genital (testis and prostate) cancer (29%) (Table I).

**Table I.** Description of selected cancer survivors in 2004/2005 showing average age, distribution of educational level and main sectors of work, working hours per week, and cancer diagnoses among persons 30–50 years old who were diagnosed with invasive cancer in 2004/2005 and who still were alive in 2014/2015 (*n*= 2*629*).

	Women		Men		Total	
	*n* (%)	Mean (SD)	*n* (%)	Mean (SD)	*n* (%)	Mean (SD)
**Population**	1675 *(64)*		954 *(36)*		2629 *(100)*	
**Age** at time for selection (2004/2005)		*42* (5.7)		*41* (6.0)		*42* (5.9)
**Level of education**						
Basic or unknown level	319 *(19)*		162 *(17)*		481 *(18)*	
Secondary	681 *(41)*		454 *(48)*		1135 *(43)*	
University, low	558 *(33)*		232 (*24)*		790 *(30)*	
University, high	117 *(7)*		106 *(11)*		223 (9)	
**Cancer site**						
Digestive organs	105 *(6)*		105 *(11)*		210 *(8)*	
Respiratory and intrathoracic organs	11 *(7)*		18 *(2)*		29 *(1)*	
Hematopoietic and reticuloendothelial tissues	36 *(2)*		51 *(5)*		87 *(3)*	
Skin (excl. malignant melanoma)	236 *(14)*		131 *(14)*		367 (*14)*	
Mesothelial and soft tissue	10 *(0.5)*		12 *(1)*		22 *(1)*	
Breast	655 *(39)*		0 *(0)*		655 *(25)*	
Female genital organs	254 *(15)*		0 *(0)*		254 *(10)*	
Male genital organs	0 *(0)*		278 *(29)*		278 *(11)*	
Urinary tract	25 *(2)*		74 *(9)*		99 *(4)*	
Eye, brain and other	138 *(8)*		105 *(11)*		243 *(9)*	
Thyroid and other endocardial	113 *(7)*		50 *(5)*		163 *(6)*	
Other (lip, bone, ill-defined, unknown/missing)	92 *(6)*		130 *(14)*		222 *(8)*	

### Labor-force participation

**Figure 1.**
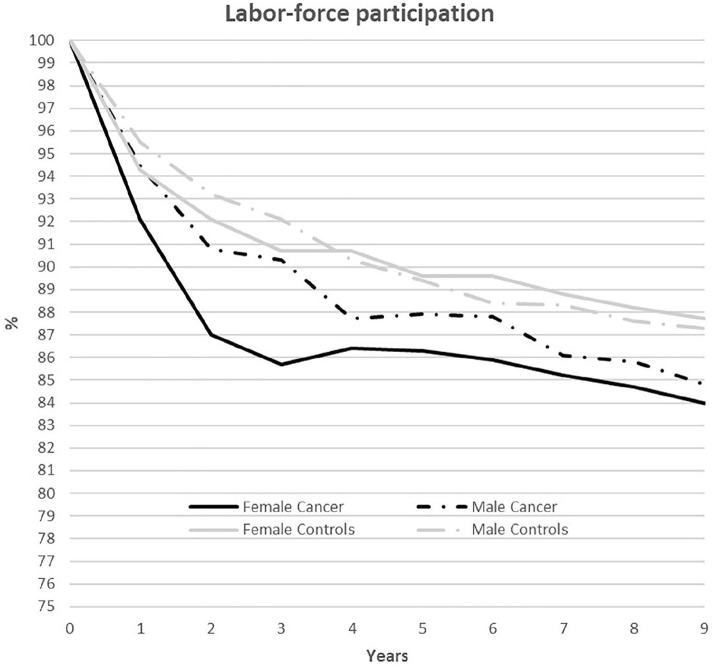
Annual employment rate (%) from 2004/2005 (T_0_) to 2013/2014 (T_9_) among working persons who survived cancer (*n*=2629) and a control group matched on gender, employment, age, and education (*n*=5258).

Figure 1 presents employment rates of PSC and controls, stratified by gender. In all groups of PSC and controls, labor-force participation decreased (*p*<0.001) over the 9-year period, and in all years fewer PSC of both genders than controls worked. Labor-force participation decreased more among female PSC than female controls, from 100% at T_0_ (similar for all groups) to 83.9% at T_9_ for PSC and 87.7% for controls (Figure 1). The difference in rate between female groups was largest at T_2–3_ (2007/2008), at 5.1% (*p*<0.001), whereas at 3.3–4.0% it was relatively stable from T_4_ to T_9_ (*p*<0.002).

Labor-force participation was lower among male PSC than male controls from T_1_ to T_9_, but the difference was significant only at T_2_ (*p*=0.019) and T_4_ (*p*=0.039). Overall, male PSC dropped to 84.8% at T_9_ and controls to 87.3%.

The employment rate was significantly lower among female than among male PSC in T_1_–T_3_ (*p*<0.02). No significant gender difference in employment rate was found in controls.

### Working hours

#### Full-time work

**Figure 2.**
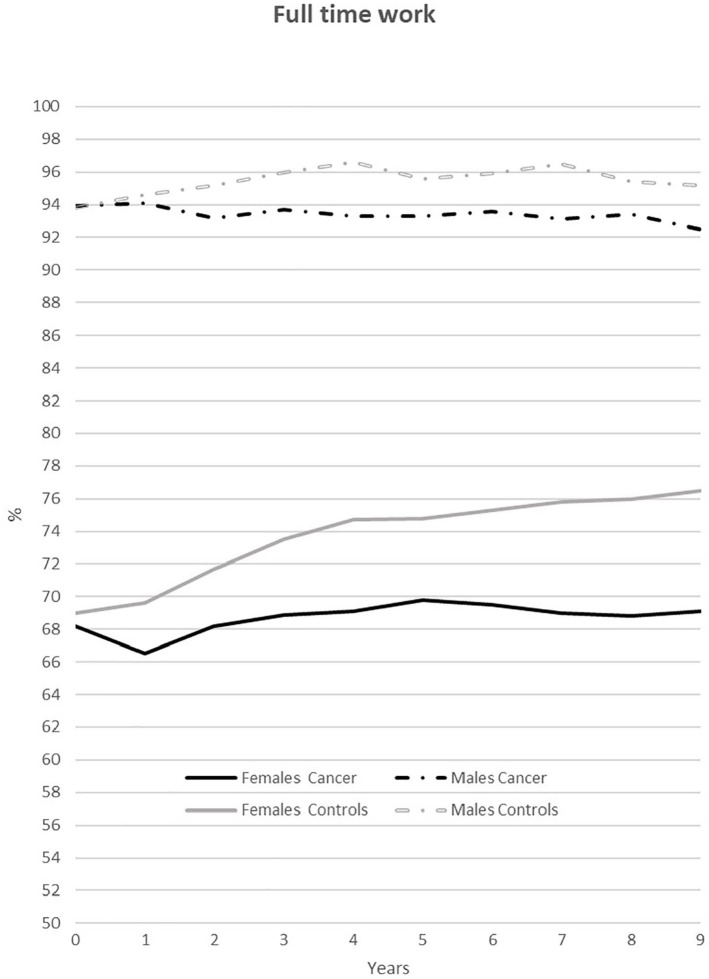
Annual employment rate (%) from 2004/2005 to 2013/2014 among persons who survived cancer and worked 30 hours or more per week (*n*=2629–2216) and a control group accordingly, matched on gender, employment, age and education (*n*=5258–4604).

Among those who remained work-ing, we found a significant reduction (*p*<0.001) in proportions of individuals working full time from T_0_–T_9_ for all groups. We also found differences in full-time work (>30 hours per week) between PSC and controls as well as by gender in both groups (Figure 2). A consistent 68–70% of female PSC worked full-time over all 9 years, whereas the proportion for female controls increased from 2005 (T_0_) to 2013/2014 (T_9_) for a significant (*p*<0.001) difference between female groups for all 9 years.The proportion of male PSC who worked full-time decreased from 93.9% at T_0_ to 92.5% at T_9_. The pattern for male controls (Figure 2) was quite similar, but the proportion working did not decrease as much as among PSC and was slightly higher (93.8–95.2%) from T_1._ The difference between male groups was significant (*p*<0.04) for T_1_–T_2_ and T_4_–T_9._

Among males (PSC and controls), the proportions in full-time work were higher for all years compared to females, and the differences between female and male PSC and between female and male controls were significant (*p*<0.001) for all years.

#### Part-time work

**Figure 3.**
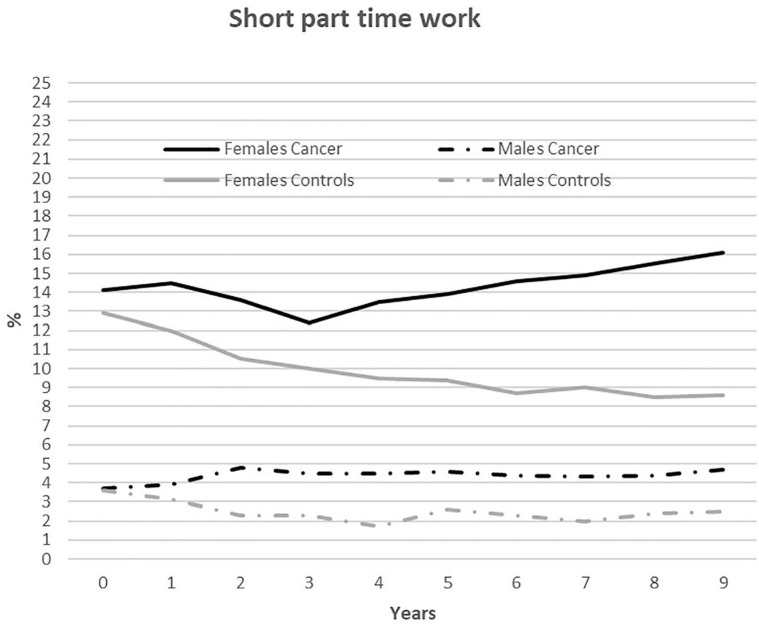
Annual employment rate (%) from 2004/2005 (T_0_) to 2013/2014 (T_9_) among persons who survived cancer and worked less than 20 hours a week (*n*=2629–2216) and a control group accordingly, matched on gender, employment, age, and education (*n*=5258–4604).

We found a significant reduction in proportions of individuals working short part-time (< 20 hours per week) from T_0_–T_9_ for female and male controls (*p*<0.002), but no significant changes in T_0_–T_9_ for the PSC groups. Figure 3 presents the results, which show several differences between PSC (both genders) and controls and between female and male PSC. The proportion of female PSC who worked short part-time first decreased to 12.4% at T_3_, before it increased to 16.1% at T_9_, whereas the proportion among female controls decreased from 13% at T_0_ to 8.6% T_9_. The differences between female groups were significant (*p*<0.02) every year from T_1_.

For male PSC, the proportion who worked short part-time increased for the first 2 years, from 3.7% at T_0_ to 4.8% at T_3_, and stabilized higher than the controls thereafter (Figure 3). The difference between male groups was significant from T_3_ to T_9_ (*p*<0.01).

Significantly (*p*<0.001) more, and an increasing number, of female PSC worked short part-time compared to male PSC, among whom the proportion remained stable from T_2_ to T_9_. Differences in short part-time work between male and female PSC and between female and male controls were significant (*p*<0.001) every year.

There were few differences between PSC of both genders and their controls in the group that worked long part-time (20–29.9 hours per week). Among the female groups, proportions decreased from approximately 19% at T_0_ to 14% at T_9_, and among male groups proportions remained stable at 2–3% (data not shown).

### Job change

**Figure 4.**
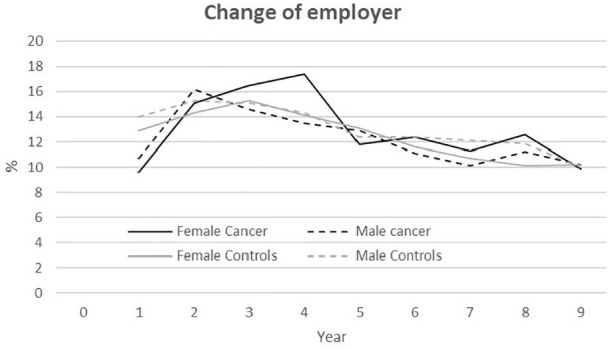
Annual rate (%) in employer changes from the first year after cancer diagnosis for PSC; 2005/2006 (T_1_) to 2013/2014 (T_9_) among persons who survived cancer (*n*=2455–2216) and a control group accordingly, matched on gender, employment, age, and education (*n*=4981–4604). Because no data were available for the year before 2004/2005, changes were impossible to measure from the time of diagnosis (T_0_) to T_1_.

As Figure 4 shows, 10–17% of participants (both PSC and controls) changed employer every year during follow-up, with a steady decrease from T_2_ to T_9_; the only exception was female PSC, who increased changes of employer from T_2_ to T_4_ and changed employer significantly more than female controls at T_1_, T_4_, and T_8_ (*p*<0.01).

Except for T_1_ (*p*=0.012), there were no significant differences between male PSC and controls regarding change of employer. Differences between female and male PSC were small, and only significant at T_4_ (*p*=0.010).

### Education

**Figure 5.**
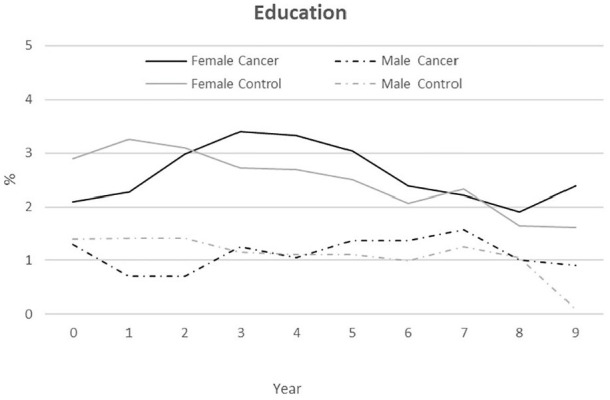
Annual rate (%) in education from 2005/2006 (T_1_) to 2013/2014 (T_9_) persons who survived cancer (*n*=2629) and a control group (*n*=5258).

About 2–3% of female PSC and controls attended education over almost the entire follow-up period, and approximately 1% of male PSC and controls (Figure 5). The differences between PSC (both genders) and controls were not significant, whereas the difference between female and male PSC was significant for all years (*p*<0.05) except T_8_ (*p*=0.081). The pattern was basically the same among male and female controls.

## Discussion

Over 9 years, PSC of both genders fell out of work more often than controls, and female PSC dropped out of work significantly more often than controls of both genders (T_1_–T_9_) and male PSC (T_1_–T_3_). Compared to controls and male PSC, significantly fewer female PSC worked full-time, and this difference increased steadily over time. Also, male PSC worked full-time less often than male controls. Female PSC increased their participation in short part-time work, and the difference between them and controls in this regard increased for the last 6 years. The proportion of male PSC working short part-time was lower than that of female PSC but higher than that of male controls. There were few significant differences among groups regarding job change or being in education.

The reduction in labor-force participation among PSC found here confirms earlier research [3, 9, 20], but we show a higher proportion of PSC returning to work than most other studies. Approximately 87% of PSC in our study were still in the workforce 5 years after diagnosis, decreasing to approximately 85% after 9 years (Figure 1), whereas international and earlier Norwegian studies show about 60–80% of PSC returning to work [3, 9, 20]. Decline of working ability has been suggested to be much higher among PSC than among non-cancer groups [9], seemingly due to lower work ability because of cancer-treatment-related late effects [6, 7]. Longer sickness-related absence periods are also a prognostic factor for falling out of work [26, 27]. Cancer treatment often leads to longer absence periods [6], which may also explain some of the difference between PSC and controls in the present study (Figure 1). Nonetheless, that does not explain the lower discrepancy in the present study than previously.

The reasons for the relatively high workforce participation and the small difference between PSC and controls here may have other explanations. Cancer treatment in Norway presumably does not lead to fewer late effects than in other developed countries; a more plausible explanation could be that we have included a group of healthier PSC by excluding those who had more than one cancer diagnosis. Also, PSC who died during follow-up were not included in the analyses; if they had been included, the number of PSC would have been raised by approximately 50% and a higher proportion of PSC would probably have dropped out of work compared to controls. Also, the participants were rather young, with a mean age of 41–42 years at T_0_. Norwegian reports have documented that in general, labor-force participation in Norway peaks around the age of 40–50 years [23], at which point the employment rate for both genders starts decreasing and sickness absences and people taking disability pensions start increasing [23, 24]. This may also explain the decrease in labor-force participation among the controls in the present study. Consequently, the present results indicates that even if many PSC manage to participate in the labor force over a time span of 9 years, whether cancer is more or less invasive may also have more impact on people than general life changes, even in this population of PSC that likely excluded those with more severe cancers. More research is required, however.

The present study also found considerable gender differences. In a Norwegian registry study using data from 1999–2004, Torp et al. [20] found lower labor-force participation among female PSC compared to both female controls and to male PSC, which resembles the results of the current study. Interestingly, the same study showed that the difference between women and men increased steadily over the 5 years following the cancer diagnosis, indicating the difference between genders might become even larger after 5 years of survival. Our results do not, however, confirm such an increased difference. Still, the difference between women and men in labor-force participation was pronounced in all years, especially regarding the difference in working hours.

Gender differences in working patterns after cancer have been found before [7, 19, 20, 28]. Being a man has been shown to be a prognostic factor for RTW [7, 13], and working hours have tended to be reduced after cancer especially among women [2, 9]. The results of the present study support those earlier results by showing that female PSC were working less full-time and more short part-time compared to controls and male PSC. Marino et al. [7] found that married men returned to work faster than married women, and a “double-burden effect” for women has been discussed in general [29, 30]. The differences between women and men found in the present study can therefore be connected to perceived family obligations among female PSC, who may feel more responsibility to balance family and work and may therefore reduce working hours or quit working more readily than men. The men, in contrast, may feel more obliged to provide the family’s main income and therefore may also be more persistent in their efforts to return to full-time work. Consequently, a “double-burden” effect for women [30] in combination with late-effects influencing RTW after cancer [6, 9, 19] may explain why more female PSC in the present study quit work, reduced full-time work, and increased short part-time work, compared to the controls and males.

Our results regarding job changes and education did not show any big differences between PSC and controls. Women (PSC and controls) engaged in education slightly more often than men, but the differences were significant in some but not all years, and more research is necessary to reveal if there are different choice paths after cancer between female and male PSC in their RTW processes.

In this study we have compared long-term RTW patterns of female and male PSC with controls. This knowledge may be informative when planning future services and support systems, because the RTW patterns revealed that several years may be needed before RTW is achieved after cancer, and that women seemed to downgrade working hours or quit working more often than men.

### Strengths and limitations

The major strengths of this study are the long-term monitoring of reliable data from registries that included all cancer cases in Norway and the comparison of PSC with matched controls that were not diagnosed with cancer.

As for possible limitations, first, men diagnosed with cancer tend to be older than women diagnosed with cancer [28]. This may explain why women represent 64% of PSC in the present study. Next, the selected study population that included PSC and controls who did not die during the 9 years of follow-up may have led to a too positive interpretation of the proportions of PSC returning to work after cancer, compared to other studies that include all working PSC. Consequently, it is essential to interpret results per the groups investigated and not generalize to the total population of PSC.

The primary aim of this study was to describe the working patterns of PSC and not to investigate causality. We monitored groups and all variables each year and did not follow individual trajectories. Individuals could therefore leave and return to different categories and groups without being monitored. For example, in the categories of working hours, we could not notice or discuss the directions of changes, for instance explain a decrease of a proportion in one category compared to another. Future studies following individual trajectories may explore how the changes in patterns develop.

For a better understanding of why female and male PSC differ from groups not having the experience of cancer, more research on data that identify changes in type of work and education is recommended. Furthermore, by exploring the causality behind working patterns and by including demographic and socioeconomic variables such as income and sick-leave patterns, more light can be shed on how to strengthen RTW processes after cancer in the future. It would also be valuable to perform cross-disease investigations to determine if, and how, the RTW patterns differ between diverse cancer types.

## Conclusions

This controlled registry study among working adults who survived for more than 10 years after cancer diagnosis confirms that cancer and its treatment can lead to persistent lower labor-force participation and the timeframe of this effect goes beyond the first 5 years after the cancer diagnosis for both genders. In addition to falling out of the labor force more often than male PSC, more female PSC also reduce their working hours, and the drop and difference increased over the whole 9-year period. More in-depth research investigating causality and long-term working patterns of persons living with a previous cancer diagnosis is recommended.

## Supplemental Material

SJP953330_Supplemental_material – Supplemental material for Labor-force participation and working patterns among women and men who have survived cancer: A descriptive 9-year longitudinal cohort study Click here for additional data file. Supplemental material, SJP953330_Supplemental_material for Labor-force participation and working patterns among women and men who have survived cancer: A descriptive 9-year longitudinal cohort study by Birgit Brusletto, Roy A. Nielsen, Harald Engan, Line Oldervoll, Camilla M. Ihlebæk, Nina Helen Mjøsund and Steffen Torp in Scandinavian Journal of Public Health
