# Supplementary material for: Labor-force participation and working patterns among women and men who have survived cancer: A descriptive 9-year longitudinal cohort study
Source: Scand J Public Health. 2020 Sep 4;49(2):188–96. doi: 10.1177/1403494820953330 (PMC7917565; doi:10.1177/1403494820953330)
Supplement: SJP953330_Supplemental_material – Supplemental material for Labor-force participation and working patterns among women and men who have survived cancer: A descriptive 9-year longitudinal cohort study [file SJP953330_Supplemental_material.pdf]

Supplemental material for "Labor-force participation and working patterns among women and men who have survived cancer: A descriptive 9-year longitudinal cohort study" by Birgit Brusletto, Roy A. Nielsen , Harald Engan , Line Oldervoll, Camilla M. Ihlebæk, Nina Helen Mjøsund and Steffen Torp in Scandinavian Journal of Public Health; Table 2-4.

Table 2. Annual labor-force participation rate (%) among Norwegians aged 30-50, alive in 2015, who were employed when diagnosed with cancer in 2004/2005 (n = 2629) and a comparison with a control group matched on gender, employment, age, and education level (n = 5258).

	**Female PSC** *n* **= 1,675**		**Females Controls** *n* **= 3,350**		**Males PSC** *n* **= 954**		**Males Controls** *n* **= 1,908**		**Female PSC versus female controls**	**Male PSC versus male controls**	**Female PSC versus male PSC**	**Female controls versus male controls**
**Year**	*%*	*95% Confidence Intervals*	*%*	*95% Confidence Intervals*	*%*	*95% Confidence Intervals*	*%*	*95% Confidence Intervals*	*P-value*	*P-value*	*P-value*	*P-value*
T_0	100	-	100	-	100	-	100	-	*1*	*1*	*1*	*1*
T_1	92.1	[90.8, 93.4]	94.4	[93.6, 95.1]	94.5	[93.1, 96.0]	95.5	[94.6, 96.5]	*0.002*	*0.240*	*0.016*	*0.063*
T_2	87.0	[85.4, 88.7]	92.1	[91.1, 93.0]	90.8	[88.9, 92.6]	93.2	[92.1, 94.4]	*<0.001*	*0.019*	*0.004*	*0.119*
T_3	85.7	[84.1, 87.4]	90.8	[89.8, 91.5]	90.3	[88.3, 92.1]	92.1	[90.9, 93.3]	*<0.001*	*0.098*	*0.001*	*0.106*
T_4	86.4	[84.8, 88.1]	90.7	[89.7, 91.7]	87.7	[85.7, 89.8]	90.3	[88.9, 91.6]	*<0.001*	*0.039*	*0.347*	*0.580*
T_5	86.3	[84.7, 88.0]	89.6	[88.5, 90.6]	87.9	[85.9, 90.0]	89.4	[88.0, 90.7]	*0.001*	*0.256*	*0.237*	*0.801*
T_6	85.9	[84.2, 87.5]	89.6	[88.5, 90.6]	87.8	[85.8, 89.9]	88.4	[87.0, 90.6]	*<0.001*	*0.652*	*0.150*	*0.203*
T_7	85.2	[83.6, 87.0]	88.8	[87.7, 89.8]	86.1	[83.9, 88.3]	88.3	[86.8, 89.7]	*<0.001*	*0.093*	*0.573*	*0.571*
T_8	84.7	[82.9, 86.4]	88.7	[87.6, 89.8]	85.8	[83.6, 88.1]	87.6	[86.2, 89.1]	*<0.001*	*0.181*	*0.409*	*0.661*
T_9	83.9	[82.2, 85.7]	87.7	[86.6, 88.8]	84.8	[82.5, 87.1]	87.3	[85.8, 88.8]	*<0.001*	*0.063*	*0.560*	*0.252*
**Change***	**Diff.**		**Diff.**		**Diff.**		**Diff.**					
T_0-T_9	16.1	[14.3, 17.8] ****	12.3	[11.1, 13.4] ****	15.2	[12.9, 17.5] ****	12.7	[11.2, 14.2] ****	-	-	-	-

*Comparison start/end of follow-up (T_0-T_9), paired t-test
**p>0.05,
***p<0.05
**** p<0.001

Table 3. Annual labor-force participation rate (%) from 2004/2005 to 2013/2014 among persons who survived cancer and worked 30 hours or more per week (*n*=2629-2216) and a control group accordingly, matched on gender, employment, age, and education (*n*=5258-4604).

	**Female PSC** *n* **= 1,675-1,405**		**Female Controls** *n* **= 3,350-2,938**		**Male PSC** *n* **= 954-809**		**Male Controls** *n* **= 1,908-1,666**		**Female PSC versus female controls**	**Male PSC versus male controls**	**Female PSC versus male PSC**	**Female controls versus male controls**
**Year**	*%*	*95% Confidence Intervals*	*%*	*95% Confidence Intervals*	*%*	*95% Confidence Intervals*	*%*	*95% Confidence Intervals*	*P-value*	*P-value*	*P-value*	*P-value*
T_0	68.2	[65.9, 70.4]	69	[67.4, 70.5]	93.9	[92.4, 95.4]	93.8	[92.7, 94.8]	*0.5761*	*0.869*	*<0.001*	*<0.001*
T_1	66.5	[64.2, 68.9]	69.6	[68.0, 71.2]	94.1	[92.6, 95.7]	94.6	[93.6, 95.7]	*0.036*	*0.030*	*<0.001*	*<0.001*
T_2	68.2	[65.8, 70.6]	71.7	[70.1, 73.3]	93.2	[91.5, 94.9]	95.2	[94.2, 96.2]	*<0.001*	*0.001*	*<0.001*	*<0.001*
T_3	68.9	[66.5, 71.3]	73.5	[71.9, 75.1]	93,7	[92.1, 95.4]	96	[95.1, 96.9]	*0.001*	*0.096*	*<0.001*	*<0.001*
T_4	69.1	[66.7, 71.5]	74.7	[73.1, 76.2]	93,3	[91.6, 95.0]	96.6	[95.7, 97.4]	*<0.001*	*<0.001*	*<0.001*	*<0.001*
T_5	69.8	[67.5, 72.2]	74.8	[73.2, 76.3]	93,3	[91.6, 95.0]	95.6	[94.6, 96.6]	*<0.001*	*0.015*	*<0.001*	*<0.001*
T_6	69.5	[67.1, 71.9]	75.3	[73.8, 76.9]	93,6	[91.9, 95.2]	95.9	[95.0, 96.9]	*<0.001*	*0.01*	*<0.001*	*<0.001*
T_7	69	[66.6, 71.4]	75.8	[74.2, 77.3]	93,1	[91.3, 94.8]	96.5	[95.6, 97.4]	*<0.001*	*<0.001*	*<0.001*	*<0.001*
T_8	68.8	[66.3, 71.2]	76	[74.5, 77.6]	93,4	[91.7, 95.1]	95.4	[94.4, 96.4]	*<0.001*	*0.037*	*<0.001*	*<0.001*
T_9	69.1	[66.7, 71.5]	76.5	[75.0, 78.1]	92,5	[90.6, 94.3]	95.2	[94.2, 96.2]	*<0.001*	*0.006*	*<0.001*	*<0.001*
**Change***	**Diff.**		**Diff.**		**Diff.**		**Diff.**					
T_0-T_9	10.2	[7.5, 12.8] ****	1.8	[0.1, 3.6] ***	15.5	[12.8, 18.2] ****	10.6	[8.9, 12.3] ****	-	-	-	-

*Comparison start/end of follow-up (T_0-T_9), paired t-test, n=T_0
**p>0.05,
***p<0.05
**** p<0.001

Table 4. Annual labor-force participation rate (%) from 2004/2005 to 2013/2014 among persons who survived cancer and worked less than 20 hours a week (*n*=2629-2216) and a control group accordingly, matched on gender, employment, age, and education level (*n*=5258-4604).

	**Female PSC** ***n* = 1,675-1,405**		**Female Controls** ***n* = 3,350-2,938**		**Male PSC** ***n* = 954-809**		**Male Controls** ***n* = 1,908-1,666**		**Female PSC versus female controls**	**Male PSC versus male controls**	**Female PSC versus male PSC**	**Female controls versus male controls**
**Year**	*%*	*95% Confidence Intervals*	*%*	*95% Confidence Intervals*	*%*	*95% Confidence Intervals*	*%*	*95% Confidence Intervals*	*P-value*	*P-value*	*P-value*	*P-value*
T_0	14.1	[12.4, 15.8]	13.0	[11.8, 14.1]	3.7	[2.5, 4.9]	3.6	[2.8, 4.5]	*0.278*	*0.944*	*<0.001*	*<0.001*
T_1	14.5	[12.7, 16.2]	12	[10.8, 13.1]	3.9	[2.6, 5.1]	3.1	[2.3, 3.9]	*0.016*	*0.269*	*<0.001*	*<0.001*
T_2	13.6	[11.8, 15.3]	10.5	[9.5, 11.6]	4.8	[3.4, 6.3]	2.3	[1.6, 3.0]	*0.003*	*<0.001*	*<0.001*	*<0.001*
T_3	12.4	[10.7, 14.1]	10	[9.0, 11.1]	4.5	[3.1, 5.9]	2.3	[1.6, 3.0]	*0.017*	*0.002*	*<0.001*	*<0.001*
T_4	13.5	[11.8, 15.3]	9.5	[8.5, 10.6]	4.5	[3.1, 6.0]	1.7	[1.1, 2.4]	*<0.001*	*<0.001*	*<0.001*	*<0.001*
T_5	13.9	[12.1, 15.7]	9.4	[8.4, 10.4]	4.6	[3.2, 6.1]	2.6	[1.8, 3.3]	*<0.001*	*0.006*	*<0.001*	*<0.001*
T_6	14.6	[12.8, 16.4]	8.7	[7.7, 9.7]	4.4	[3.0, 5.8]	2.3	[1.6, 3.0]	*<0.001*	*0.004*	*<0.001*	*<0.001*
T_7	14.9	[13.1, 16.8]	9	[8.0, 10.1]	4.3	[2.9, 5.6]	2	[1.3, 2.7]	*<0.001*	*0.001*	*<0.001*	*<0.001*
T_8	15.5	[13.6, 17.4]	8.5	[7.5, 9.5]	4.4	[3.0, 5.8]	2.4	[1.7, 3.1]	*<0.001*	*0.006*	*<0.001*	*<0.001*
T_9	16.1	[14.2, 18.1]	8.6	[7.6, 9.6]	4.7	[3.2, 6.2]	2.5	[1.7, 3.2]	*<0.001*	*0.003*	*<0.001*	*<0.001*
**Change***	**Diff.**		**Diff.**		**Diff.**		**Diff.**					
T_0-T_9	0.5	[-1.6, 2.6] **	5.4	[4.2, 6.8] ****	-0.3	[-1.8, 1.2] **	1.5	[0.1, 2.4] ***	-	-	-	-

*Comparison start/end of follow-up (T_0-T_9), paired t-test, n=T_0

**p>0.05,

***p<0.05

**** p<0.001
